# “He told me my pain was in my head”: mitigating testimonial injustice through peer support

**DOI:** 10.3389/fpain.2023.1125963

**Published:** 2023-05-22

**Authors:** Marie Vigouroux, Gillian Newman, Kristina Amja, Richard Bruce Hovey

**Affiliations:** ^1^ Department of Integrated Studies in Education, Faculty of Education, McGill University, Montreal, QC, Canada; ^2^ Institute for Gender, Sexuality and Feminist Studies, Faculty of Arts, McGill University, Montreal, QC, Canada; ^3^ Curvy Girls, Toronto, ON, Canada; ^4^ Faculty of Dental Medicine and Oral Health Sciences, McGill University, Montreal, QC, Canada

**Keywords:** peer support, testimonial injustice, pediatric pain, scoliosis, applied philosophical hermeneutics, qualitative research

## Abstract

**Introduction:**

Women with disabilities are exposed to sexism and ableism, earn less income, and work in exceptionally challenging conditions compared to women without disabilities and men with or without disabilities. Adolescent girls living with scoliosis may begin experiencing this compounding bias during their encounters with healthcare from the moment they start noticing differences in their bodies. Being significantly more likely than boys to progress to a curve angle where painful treatment such as bracing or spinal fusion surgery is required, adolescent girls living with scoliosis are therefore more likely to experience chronic pain. The long-term impact of pain and pain-related stigma includes lower educational attainments, decreased vocational functionality, and social impairments in adults after having experienced chronic pain in adolescence.

**Approach:**

In this article, the authors will explore the effects and mechanisms of gender-specific peer support in disrupting this trajectory to adverse outcomes. Through individual interviews consisting of open-ended questions, the researchers gathered narrative data from *Curvy Girls* members, a community-based peer support group for girls and young women living with scoliosis. The data was analyzed using an applied philosophical hermeneutics approach, with intersectionality and testimonial injustice as their framework.

**Findings:**

They found that the study participants had their pain narratives reinterpreted by the adults in their lives, including their parents and healthcare practitioners, leading them to question and doubt their own experiences.

**Discussion:**

These negative outcomes were mitigated through the peer support they received and offered from *Curvy Girls*. Participants reported having gained confidence and a sense of belonging after they joined this group, allowing them to better cope with their condition more effectively in different facets of their lives.

## 1. Background

Adolescent girls living with scoliosis face several challenges and difficulties related to and compounded by their health status and gender. The most common spinal disorder amongst adolescents, scoliosis is a condition where a lateral curve develops in the spine, resulting in low back pain in up to 85% of cases (1, 2). In this article, the term “scoliosis” will be used throughout this paper as an umbrella term to refer to all groups and sub-groups of the condition. As they enter adolescence, girls are more likely to develop scoliosis than boys and are significantly more likely than boys to progress to a curve angle where treatment such as bracing or spinal fusion surgery is required (1). Some adolescents with undiagnosed scoliosis will seek care when they or their loved ones notice a hump along their ribs or their back or a lack of symmetry in their hips, while others will seek care regarding their back pain (2).

Current treatments for scoliosis include bracing, a practice where the patient wears a corset-like orthotic device for at least eighteen hours per day to slow the development of their spinal curvature and attempt to correct it (3, 4). While this non-surgical approach has been shown to be effective, it is also associated with decreased pulmonary function, chronic pain, and psychological distress (4–6). In this article, “chronic pain” will be used as an umbrella term to include any persistent or recurrent pain lasting more than three months (7). If bracing fails to slow curve progression or if the condition is too advanced to attempt bracing, a surgical intervention may be required (3). Described as the standard for correcting scoliosis for the past century, spinal fusion is an extensive and invasive surgery that entails straightening the spine and inserting surgical pins and rods to ensure long-term correction (8, 9). Despite its extensiveness, spinal fusion surgery is a safe and effective surgical approach to treating scoliosis, with low rates of adverse outcomes (8, 10). Still, persistent and chronic post-operative pain is the leading complication for this intervention, with 17% of patients reporting moderate to severe pain five years post-surgery, potentially impacting self-image and mental health (8, 9).

Given that scoliosis and its treatments are associated with chronic pain, it is essential to understand the negative social outcomes of experiencing it in adolescence. A systematic review evaluated nine studies that investigated peer relationships of children and adolescents living with different chronic pain conditions. The review found that the majority of these studies noted negative outcomes in peer relationships for this population (11). Four out of five studies that compared children and adolescents living with chronic pain to healthy controls found higher instances of peer relationship difficulties in those living with chronic pain (11). Additionally, the review found that this population has fewer peer relationships (including reciprocal friendships and best-friendships) than healthy controls. Adolescents living with painful conditions were also found more likely to be described as isolated and sensitive, as well as “less popular among their peers, and less likely to exhibit leadership skills.” (11) Negative social outcomes will follow these children and adolescents into adulthood: the long-term impact of experiencing chronic pain in adolescence includes lower educational attainments, decreased vocational functionality, and social impairments in young adults (12, 13).

Adolescents living with chronic pain are also subjected to pain-related stigma by medical professionals, education personnel, peers, and family members (14, 15). Parental invalidation has been linked to negative mental health outcomes such as self-harm and suicide (16). Children and adolescents experiencing pain invalidation from parents, educators, friends, or health care providers can learn to suppress pain expression to escape pain-related stigma in an attempt to mitigate social exclusion (17). This behavior can lead to decreased health outcomes if their pain is not investigated or treated by a health care provider. This is especially troubling for adolescent girls, who have been found more likely than adolescent boys to experience chronic pain and report higher pain intensity and dismissal from health care providers (18). This gender difference follows adolescent girls into adulthood, as adult women reporting chronic pain are also more likely than men to experience pain dismissal, be undertreated, receive nonspecific diagnostic, receive fewer follow-ups, and be told that their condition is the result of a psychological condition (18).

Patient-facing literature suggests that people living with chronic pain could benefit from joining peer support groups to learn how to better manage and cope with their condition (12). Globally, peer support—defined as peer-to-peer relationships not necessarily within the context of a support group—has been associated with decreased risks of bullying and victimization, as well as significantly reduced rates of mental health issues such as anxiety and suicidal ideation (19, 20). It is also associated with higher odds of engaging in healthy behaviors such as physical activity (21). A review of different online peer support resources found that despite a growing number of these platforms and resources available to youth living with chronic pain, there is a paucity of academic literature on the outcomes and mechanisms of peer support groups for this population (22). While some studies have found positive outcomes for specific peer support programs for youth with chronic illness and chronic pain, none focused on adolescent girls with scoliosis (23, 24). *Curvy Girls* is a community-based peer support group for girls living with scoliosis. A play on words about the spinal curvature of the participants hinting at the unrealistic feminine beauty standards promoted in traditional and social media, *Curvy Girls* aims to connect girls living with scoliosis with each other through local groups and social media (25, 26). This study explores the role and mechanisms of the peer support group in disrupting the participants' trajectories toward social isolation.

## 2. Theoretical framework

From the information in the previous section, it becomes clear that special specific attention must be paid to the experiences of adolescent girls living with scoliosis. As girls living with a chronic health condition, they are at increased risk of earning less income and working in worse conditions in adulthood than women without disabilities and men with or without disabilities (27). This risk highlights the importance of using intersectionality as part of the framework for understanding the narratives presented in this article. Intersectionality is a term originally coined by Crenshaw in 1991 to explain American Black women's social location making them uniquely vulnerable to violence and discrimination, even compared to Black men and white women (28). Crenshaw argues that one's social location is not made up of separate components that add to one another but of complex identities that compound the likelihood of experiencing systemic oppression. Over the past thirty years, the term has been expanded to include other factors that may lead to restricting one's social freedoms or access to care and services, such as age, disability, class, sexual orientation, an extended understanding of gender identity, etc (29–31).. While our current understanding of the factors included in intersectionality has in some ways outgrown Crenshaw's original definition and purpose, its mechanism remains the same: layered identities interact with each other to create a unique social location that will influence the social, financial, and health outcomes of people at this intersection. In the past ten years, there has been a growing body of research exploring inequities in healthcare for individuals at varying intersections of race, gender, and disability, showing a dire need to translate this research to clinical practice (32–34).

Another building block of the theoretical framework employed in this article is Fricker's concept of testimonial injustice (35). Fricker defines testimonial injustice as a credibility deficit derived from identity prejudice, where the hearer of testimony has their perception of the speaker's identity and credibility distorted by prejudices living in the social imagination (35). The hearer, through their evaluation of the speaker's epistemic trustworthiness, based upon their biased perception of the speaker's competence or sincerity, wrongfully determines that the speaker's knowledge is not to be trusted (35). The immediate harm of testimonial injustice is the harm to the speaker in their capacity as a knower, a direct undermining of their capacity to reason, their human value, and their humanity (35). The speaker will likely carry this dehumanizing experience or experiences and may begin to doubt their testimony and their capacity as a knower (35). However, it must be acknowledged that testimonial injustice also diminishes the knowledge of the hearers who dismiss the speakers' testimonies as they do not integrate the knowledge offered by the speakers they dismiss. In the context of clinical care, testimonial injustice may result in diminished quality of care through delayed diagnosis or misdiagnosis and patients' distrust in healthcare systems and workers (36). While many articles exploring testimonial injustice tend to focus on one axis of identity, such as gender, disability, or race, Patricia Hill Collins highlights the importance of intersectionality in testimonial injustices:

Epistemic oppression constitutes a core defining feature of intersecting systems of power, a fact made visible by intersectionality's border-crossing from epistemic communities of social activism to those defining academic norms. The construct of epistemic oppression identifies how epistemology constitutes a structuring dimension of social injustice beyond actual ideas of racism, sexism and similar ideological system (37).

Despite efforts to incorporate patients’ narratives into the diagnosis and care process, the asymmetrical power structures between physicians and patients allow testimonial injustice to occur and perdure (38, 39).

Finally, the third building block of the framework used in this article is the use of applied philosophical hermeneutics in conjunction with intersectionality and testimonial injustice to attempt to explore the data at an individual and systemic level. As described in the section below, applied philosophical hermeneutics is a research approach that allows for deep exploration of and reflection on the topic at hand. This approach is derived from Gadamer's rejection of the scientific method as the best or only approach to truth and calls for a socially-constructed epistemology (40, 41). Gadamer's philosophical hermeneutics have important applications in health care, particularly in understanding, bettering, and fostering relationships between patient and physician (42). While feminists have historically preferred Foucault to Gadamer, frameworks that combine feminist theories and philosophical hermeneutics exist and thrive (43). Buker describes different ways in which feminists and feminist theorists can use philosophical hermeneutics to enrich their approach to research (44). She concludes that employing philosophical hermeneutics can aid feminist theorists in remaining self-reflective in their practices by allying philosophical hermeneutics' drive to understand and (re)interpret tradition with feminism's determination to build more just social systems (44). As such, this article will be an attempt to ally applied philosophical hermeneutics to the feminist theories of intersectionality and testimonial injustice to explore the experiences of disabled teenage girls in peer support groups.

## 3. Research approach

This study was approved by the McGill University Research Ethics Office of the Faculty of Medicine and Health Sciences (project A06-B44-19B). Recruitment occurred with the help and support of *Curvy Girls'* board of directors, who advertised the study within its public and private social media groups, as well as local support groups. To be eligible to participate in this study, participants must (1) be fourteen years of age or older, (2) be a current or past *Curvy Girls* member, (3) speak French or English, and (4) currently be living in Canada. *Curvy Girls* members interested in participating in the study would contact the research team, who assessed their eligibility and went through informed consent procedures. Participants who were above eighteen years of age signed the consent form themselves, while participants who were between the ages of 14 and 18 had their parent or guardian sign the consent form. The consent form also included a space dedicated to capturing the assent of a minor.

The narrative data were collected through open-ended semi-structured interviews with sixteen participants between April 15, 2020, and October 28, 2020. All interviews were audio-recorded and then transcribed verbatim. At the end of each interview, participants chose a pseudonym to assign to their personal narrative for publication purposes. Interview questions were planned in a funnel format to encourage participants to bring up any aspect of the question that is most important to them and discuss it in their own words (41). The first questions to the participants were broad and open-ended, such as “Could you tell me the story of your scoliosis diagnosis?”, inviting them to direct the conversation to any aspect of the question that is most relevant to their lived experience. This interview strategy has been found to signal to participants that their narratives were welcomed and encouraged thus generating rich and highly interpretable narrative data (41). Only once the narrative data was collected did the interviewers move on to more specific questions to cover some aspects of the research questions that the participants had not included in their answers to the exploratory questions such as “Do your friends or family members treat you differently?” Questions were formulated in a language appropriate and understandable to adolescent girls and young women in conjunction with *Curvy Girls'* board of directors.

The narrative data was analyzed using applied philosophical hermeneutics, a branch of Gadamerian hermeneutics (41). In this interpretive research approach, researchers engage dialogically with participants during the research interview, which becomes a transformational moment for both the interviewer and the interviewee (45). Once the interviews are transcribed, they become the starting point for the hermeneutic circle, where the researchers go from reflecting on parts of the text (the transcribed interviews) to reflecting on the whole and back to the parts (46). This approach is meant to center the experiences of the research participants through a hermeneutic wager (47, 48). Guided by the research topic rather than strict adherence to a strict research *method*, applied philosophical hermeneutics is a research *approach* that favors philosophical praxis (49, 50). Such lack of a predefined method may be unfamiliar, unnerving, and even alarming to those in the fields of health and social sciences, in which specific and reproducible research techniques have become synonymous with academic rigor (41). However, through an unmatched adaptability that has earned it the moniker of “unmethod,” applied philosophical hermeneutics “is constituted through an in-depth philosophical understanding of what comprises rigorous hermeneutic research and what does not” (41).

As such, applied philosophical hermeneutics lends itself well to incorporating feminist theories (such as intersectionality and testimonial injustice discussed above). Indeed, one of the main arguments of Gadamer is that language is what allows us to situate ourselves in the world (40). Heckman argues that Gadamer's approach to knowledge, through its rejection of positivism, requires “a reconceptualization of human knowledge itself” (51). This radical reconceptualization relies on a deep understanding of tradition, yet Heckman warns: “it is precisely the element of Gadamer's approach that has seemed most antifeminist—tradition—that is the most useful tool for feminist analysis” (51). By intending to (re)understand and (re)interpret texts situated in the hermeneutic tradition, Gadamerian hermeneutics offer a perfect place to investigate the epistemic and structural injustices at the core of feminist study.

Before proceeding to the research findings, the authors wish to acknowledge that while this study was initially conceived without the theories of intersectionality and testimonial injustice, they were not merely shoehorned in as an after-thought but carefully incorporated once it became clear to the researchers that they would be valuable frameworks through which to analyze the collected data. Fortunately, applied philosophical hermeneutics permits and encourages this kind of shift in approach mid-research, as it conveys a changed or renewed understanding of the topic, the ultimate goal of this research approach (41, 45, 49).

## 4. Findings

Three main findings will be discussed in this article: the pain dismissal experienced by the participants in medical settings, the belonging they began feeling once they engaged with *Curvy Girls,* which helped palliate the effects of feeling dismissed, and finally, the advocacy they were able to perform once they gained the confidence they needed.

### 4.1. Dismissal

When asked about her experience before receiving her diagnosis, Milan recounts that she had complained of back pain since she was five years old, only to either be misdiagnosed or have her concerns brushed aside as “growing pains” by the health professionals she consulted with this chief complaint. This continued well into her adolescence when she began noticing asymmetry in her body, particularly in her hips:

I kept commenting on my body and how it looked and [saying], “this is not normal.” People around me thought I was over-critical. They were just like “don't worry about it, she's just a teenager, she's picking at herself for no reason just because she's insecure.” […] There was something that was so obvious to me and people would kind of brush it off and be like “no, what are you talking about, you look fine!”

Milan witnessed her loved ones and her healthcare providers diminish the legitimacy of her health concerns to reflect what they interpreted as vanity and lack of self-confidence.

Evelyn described a similar experience when she sought care from her pediatrician as a pre-adolescent:

I've been expressing that I've been in pain since I was like eight or nine when I started doing gymnastics. They didn't really run any tests or anything, they just kind of said that because my family is known for having chronic pain, it's considered normal.

At the time of the interview, Evelyn had just turned eighteen and received her scoliosis diagnosis only months ago. She had spent her entire teenage years trying to cope with unexplained chronic pain, which had progressed so far that she could no longer attend classes in person and had been studying from home months before the COVID-19 pandemic made virtual classes the norm.

Emily was about thirteen years old when her parents took her to the hospital to seek help to manage and attenuate her lower back pain and nausea she was experiencing. The physician she met with assumed she was pregnant and that the pain and nausea were symptoms of her pregnancy. This interaction was shocking to Emily and left her feeling helpless and questioning her own perceptions:

He wasn't listening to me. It was so bizarre and so negative. […] To have people who are supposed to help you not even acknowledge it, it made you feel really stuck because it's “well then who is going to help me?” It just made me feel very helpless and very alone and just sad. It makes you feel “will I ever get better?” Then you start to question: “Am I really having this pain? Am I making this up? This person's a doctor and they're questioning me.”

Emily and her family later found out that her back pain was a symptom of her scoliosis and that her nausea was a side effect of the pain medication prescribed for her back pain. Yet, she was made to feel like these symptoms were the self-inflicted repercussions of teenage pregnancy. Knowing she was not pregnant, her only other option was to consider whether the pain she was experiencing was real.

Throughout her interview, Caroline recounted the challenges she had experienced in dealing with her orthopedic surgeon before and after her spinal fusion surgery:

I think the icing on the cake was at my six-week post-op appointment, I was in excruciating pain. They had to bring a stretcher out for me to lie down on. It was a mess. He told me my pain was in my head and I was making it up and he sent me to a psychiatrist.

Like Emily, Caroline's pain was dismissed and denied, causing her to question her own experiences. Her surgeon's decision to refer her to a psychiatrist reflects his belief that Caroline's acute pain was caused by a psychiatric condition unrelated to her spinal surgery just six weeks prior.

Willow also described encountering a physician who actively minimized and mocked her pain, even after she had received her scoliosis diagnosis:

He just talked about how [scoliosis] shouldn't cause pain and he was like “if you're in pain, you should be pretty worried because it's not normal for scoliosis to cause pain.” I don't know if he was a specialized scoliosis doctor saying that, but I remember I felt really invalidated after.

Along with the testimonies from Milan, Evelyn, Emily, and Caroline, Willow's experience of dismissal hints at a pattern where adolescent girls living with scoliosis, diagnosed or yet-to-be, are having their pain and other symptoms dismissed, minimized, or re-interpreted by the physicians from whom they seek care.

### 4.2. Belonging

The experiences of dismissal described above are in stark contrast with the feeling of belonging that participants felt when they joined *Curvy Girls*. In a world full of people who did not believe them, Curvy Girls was a physical or virtual space that accepted them as they were, believed and affirmed their experiences, and allowed them to make meaning of their condition.

As a local Curvy Girls chapter member, Dawson met with other members in person at regular intervals. She explains that the support group offered her a space unlike any other in her life at the time:

I just liked having other people to talk to about it who knew what it felt like more than like my mom and dad. Because my mom and dad, they would try to understand as well as they could, but it's different when you're talking to someone who has actually been through it as opposed to someone who hasn't physically been through it.

Dawson articulates that while she felt supported by her parents, that support was not as helpful in processing her experiences with scoliosis as the support she received from other adolescent girls living with scoliosis. That is not to say that parental support was not important to her, but that peer support fulfilled a need that parental support alone could not.

Also a member of a local *Curvy Girls* chapter, Kacey recalled feeling isolated as an adolescent girl living with scoliosis, knowing that others like her existed, but not knowing how to get in touch with them:

I felt comfortable. I didn't feel like I had to be scared. Or a sense of togetherness? I don't even know if that's a word. Before I was like “oh, there's maybe a few people that have scoliosis.” Now, I'm like “oh my gosh, we’re 20 girls in a room right now and we all have scoliosis, that's awesome.” Maybe like a sense of belonging, togetherness, or something down that line.

Kacey describes the internal shift from isolation to connection. Before being introduced to *Curvy Girls,* she believed she was one of “a few” people with her condition, which can bring up feelings of otherness and isolation. However, attending peer support group meetings allowed her to normalize her experience: during those meetings, what othered her from most people in her life made her immediately belong to the group.

Brenda, another member of a local chapter, recalls being relieved to be able to discuss bullying with her *Curvy Girls* peers:

Throughout school, a lot of people called me the “crooked girl.” It was nice to talk to these other girls that were like “nevermind it, you are beautiful the way you are and you can’t help the fact that you have a back disorder, it's not your fault.” (…) People, obviously, they saw my body, they saw me disfigured a bit. They knew something was up but instead of asking me what was wrong, they made fun of me. So, I kind of rolled with it and I was like “if you want to make fun of me, then make fun of me; I don’t need to give you a reason for me being who I am.”

Brenda's newfound ability to stand firm in her identity seems to be a direct result of the affirming words she heard at her *Curvy Girls* meetings.

Unlike the participants above, Mary had never attended an in-person or online *Curvy Girls* meeting. Her participation in the group was limited to social media. However, this did not dampen her enthusiasm:

It's nice to be a part of. You belong to a community. It's nice to have people who are in a similar position to you. Being able to answer questions is really nice because it gives meaning to a negative experience.

Mary articulates another essential component of belonging: giving back. Being able to offer guidance to her peers fulfilled another aspect of a need for social connection. Having been through spinal fusion surgery, she felt the need to share her experience with her peers who have not yet been through it.

Evelyn had been isolated from her friends and classmates even before the COVID-19 pandemic began, having to take all her high school courses remotely due to the pain she was experiencing from her undiagnosed and untreated scoliosis. Throughout the interview, she described challenging relationships with her friends and peers at school. It is no surprise that she expressed being nervous about fitting in with other *Curvy Girls* members:

Evelyn: I didn't know if I would be good in a support group, as I tend to be a really anxious person. I didn't know if I'd be able to open up, or be adequate for the group. But it seems fine so far and I would actually like to get more involved with it.

Interviewer: You weren't too sure if you would be a good fit for the group. What was the reality of it?

Evelyn: The reality was that I was. All you really have to be is a girl with scoliosis and you fit in perfectly. Everyone is so nice. I haven't had one bad interaction with people in the group.

Like Mary, at the time of the interview, Evelyn had kept her interactions with *Curvy Girls* to social media posts, asking questions about her upcoming spinal fusion surgery. Her feelings of inadequacy and fear of not being enough developed through years of dismissal, othering, and isolation were assuaged by the hospitality of *Curvy Girls* members.

### 4.3. Advocacy

Part of *Curvy Girls'* mission statement is to help their members “become leaders” (52). The interviews with participants showed that advocacy and giving back had become important in the journey of members who had gone through specific milestones such as bracing, surgery, high school graduation, etc.

While Evelyn's diagnosis was still very recent, she was already looking back on her past experiences differently and with a will to advocate for herself that she did not feel empowered to show previously. When asked what she would tell physicians who dismissed her pain if she could go back and advocate for herself, she replied:

I would honestly probably tell them to run x-rays and run any tests. I'd rather they come back negative than not have them be run at all. My curve now is like 60 degrees and then I have a 50-degree in the lumbar. There's a lot worse, but if they didn't run these x-rays, it would have gone worse. It's progressive. I would have definitely told them to run x-rays sooner.

Evelyn's experience of receiving her diagnosis in early adulthood instead of adolescence will forever color the way she will offer advice to her peers in the earlier stages of their journey. Her grief about what might have been if her pediatrician and other physicians had thoroughly investigated her expressions of pain before her curve progressed to a point requiring her to seriously consider spinal fusion surgery is palpable in this quote.

When asked what *Curvy Girls* has helped her with, Milan replied: “I think it's helped me become a better advocate for things that I believe in, especially scoliosis.” Dawson concurred: “The first [thing] for sure is I’ve been able to formulate questions much clearer than I ever used to when I was very shy, so that definitely helps a lot.” Milan and Dawson's respective experiences prior to becoming *Curvy Girls* members are present in their answers. By discussing what their support group has helped them with, participants also hint at what they previously struggled with and identify a previously unfulfilled need. Dawson and Milan needed to gain confidence in advocating for themselves and others. When Dawson mentions that she has become better at asking clear questions, she also reveals that she has gained reflexive skills in knowing what is important for her to know and understand about her medical care. *Curvy Girls* was a transformational space for both Milan and Dawson.

Caroline also discusses her own transformation when it comes to navigating her healthcare and healthcare team:

[My surgeon] would talk to my parents the whole time, as if I'm not important even though it's my body. It was incredibly frustrating and even in the beginning when I was meeting him, I was intimidated. I didn't want to say anything. I felt very little. By the end, I just felt like “this is ridiculous,” and I started to gain more confidence in talking to him and speaking my mind.

While all participants recalled at least one instance of dismissal through their healthcare journey, Caroline's relationship with her physician was particularly challenging. Seeing him speak exclusively to her parents, although the topics of discussion were her body and her condition, caused her to withdraw from the appointments. Being objectified in that way made her feel small and unimportant, intimidated to the point where she would remain silent. Then, just as Dawson gained the confidence to formulate questions that mattered to her, Caroline felt empowered to speak about her body, condition, symptoms, and questions at her regular appointments.

Riley discusses her transformative journey in terms of going from social isolation to social connection:

*Curvy Girls* was there as a support to make me feel like I wasn't alone and to help me work through challenges like after surgery, how to go to school, or feeling more comfortable with your brace in public. *Curvy Girls* definitely helped provide support in a community and definitely was a big part of my journey.

Riley articulates a key component of peer support: community and belonging as stepping stones to confidence and advocacy.

## 5. Discussion

Throughout the quotes and participant accounts presented in the previous section of this article, it became clear that the participants interviewed in this study underwent negative experiences with potential adverse outcomes that were mitigated partly by their association with *Curvy Girls*. Additionally, peer support seems to be acting as a helpful disruptor of the chronic pain trajectory.

Adolescents who experience chronic pain are more likely to miss more school days and feel socially isolated (53), leading to lower educational attainments, decreased vocational functionality, and social impairments in adulthood (13). Therefore, investigating reports of pain and attempting to diagnose the underlying condition(s) causing the pain should be a priority to alleviate not only the pain but the short- and long-term negative outcomes associated with chronic pain during adolescence. Since scoliosis is a progressive condition, timely diagnosis allows for non-surgical treatment options such as bracing to slow or stop the progression of the curve(s) during adolescence (3). Despite what is already known about the paths to the diagnosis of scoliosis and the negative outcomes of experiencing chronic pain during adolescence, this study highlights the gaps in clinical practice that fail to disrupt such trajectories. The narratives of pain dismissal and reinterpretation collected in this study led to feelings of anger, frustration, confusion, and isolation for the participants. These findings align with what is referred to in disability studies as benevolent ableism, which includes paternalistic and condescending behaviors from those attempting to help disabled people (54). Such behaviors from physicians toward their patients can have long-term adverse effects such as a life-long erosion of trust in healthcare systems and providers (36). These findings also align with previous studies on the experiences of adolescent girls and adult women living with chronic pain (18).

The data collected in this study indicate that being a member of *Curvy Girls* and engaging with other members in physical or digital spaces allowed the participants to advocate for themselves, their health, and enhance their quality of life. They gained confidence and assurance that manifests itself in their healthcare encounters by asking more questions about their symptoms and condition, insisting on receiving satisfactory answers, and speaking up during appointments when their physician and parents would usually dominate the conversation. These self-advocacy skills and techniques do not remove the short- or long-term negative outcomes of scoliosis, chronic pain, or invalidation. However, they aided the participants in feeling more connected to the people in their lives: first to the other *Curvy Girls* members, with whom they begin to share personal stories, tips and tricks, and best practices, then to themselves as they recognized that their experiential knowledge of their condition was valuable, and finally to friends and family as they disrupt the social isolation induced by their feelings of loneliness and otherness due to their condition.

Additionally, the narratives collected in this study hint at a testimonial injustice occurring between the participants and their physicians. We must first recognize the power differential between physicians and adolescent patients. Adolescent patients can have restricted access to healthcare. They must first advocate for themselves to their parents, who may be convinced enough by their child's pain narrative to bring them to seek medical care but may side with the physician who has determined the pain is not worth investigating. This dynamic was described in Evelyn's story. Her parents did not advocate for her pain to be investigated further after her pediatrician's repeated claims that her chronic pain may be genetic. As speakers, Evelyn and other participants suffered a credibility deficit compared with their physicians. Despite the extensive clinical experience of physicians that affords them their well-earned expertise in their field, adolescent girls who are not believed when they report pain are “wronged specifically in [their] capacity as a knower,” (35) when the topic at hand is their own bodies and pain perceptions.

Perhaps, then, a defining feature of a peer support group might be that, within its confines, it eliminates the testimonial injustice that has been experienced by the group's members. The participants reported feeling immediately connected to other *Curvy Girls* members by simply knowing that they also had scoliosis and were likely to face similar circumstances. As Kacey explained, knowing she was in a room with twenty adolescent girls with scoliosis gave her a profound sense of belonging. By listening to each other and treating each other's narratives as knowledge to be valued, individuals with similar intersecting identities may experience the opposite of the harm they have experienced through testimonial injustice. The active practice of valuing and validating their fellow members' narratives and experiences could be, in part, what allows *Curvy Girls* members to feel more confident in their own experiences and begin to advocate for themselves. *Curvy Girls* members are situated at a similar intersection of identities in terms of age, gender, and disability status. It must be noted that information about race and ethnicity was not collected in this study and thus cannot be assessed. Still, standing at these similar intersections allows *Curvy Girls* members to quickly form connections and engage with one another, allowing them to disrupt their feelings of social isolation.

The findings of this study are situated within a specific scope and limitations, including the number of participants, that all participants were Canadian, and were exclusively recruited from *Curvy Girls* and no other support group. This study's findings cannot be generalizedto the whole population of adolescent girls living with scoliosis or chronic pain, but they indicate that the experiences described in this article exist within this population and warrant further research. Future research possibilities include qualitative and quantitative studies with larger study samples as well as participants from other support groups and other countries. These larger studies could focus in on specific aspects of the population's experiences such as quantifying the link between mental health issues and scoliosis and the relationship between mental health and peer support groups. Another possible future research avenue includes closing the gender gap in care for adolescent girls and adult women living with chronic pain. Such research could affect the outcomes for this population and be highly transferrable to other chronic conditions.

## 6. Conclusion

Overall, the findings presented in this article indicate that adolescent girls living with scoliosis experience limited opportunities to discuss negative encounters with healthcare that include pain dismissal and testimonial injustice which may lead to negative mental and physical health outcomes. *Curvy Girls* disrupts this trajectory by creating physical and virtual spaces that cater to its members' needs for acceptance and belonging. This peer support group can be understood as a community-based educational and care environment that relies on the personal narratives of individuals with similar intersectional challenges. It assists its members in learning and practicing acceptance and advocacy, preparing them for the lifelong gender- and disability-related challenges they are likely to face. This study offers insight into the potential for peer support groups to improve their members' quality of life, warranting further research.

## Data Availability

The datasets presented in this article are not readily available because of research participants privacy. Requests to access the datasets should be directed to Richard B. Hovey, richard.hovey@mcgill.ca.
