# Supplementary material for: “He told me my pain was in my head”: mitigating testimonial injustice through peer support
Source: Front Pain Res (Lausanne). 2023 May 22;4:1125963. doi: 10.3389/fpain.2023.1125963 (PMC10239879; doi:10.3389/fpain.2023.1125963)
Supplement: Supplementary file 1 [file Datasheet1.pdf]

Interview Guide

1. Tell me about yourself.
    - Can you tell me the story of how you were diagnosed?
    - How did that feel? / How did you feel in that moment?
    - How has your life been since then?

2. Does scoliosis interfere with your social life? Friends, family, hobbies, colleagues, teachers…
    - Does it keep you from seeing your friends?
    - Do your friends treat you differently now than before?
    - Do your friends treat you differently than their other friends?
    - Does it make it harder for you to make new friends?
    - Does it keep you from doing things you like?
    - Do you usually tell people about it, or do you prefer to keep it to yourself?
    - How did it affect your family life?
    - Is it harder to do family activities?
    - Does your family treat you differently than before?
    - Does your family treat you differently than your siblings (if applicable)?

3. How was your experience with healthcare?
    - What kind of specialists or doctors did you see? (surgeon, social worker, psychologist, etc.)
    - Which one of the people you mentioned helped you the most?
    - How did they help you?
    - Can you tell me about your pain?
    - Do doctors/nurses believe you when you tell them you're in pain?
    - Can you give me an example of something that happened where someone didn't believe you were in pain?
    - How does it make you feel when doctors/nurses don't believe you?
    - How do you feel when someone asks you to rate your pain on a scale of 1 to 10?
    - What do these numbers mean to you?

- What questions would you rather be asked to rate your pain?

4. How did you hear from curvy girls?
    - How long have you been a member?
    - What did you think a meeting would be like?
    - How was it actually?
    - Can you name the top 3 things that CG has helped you with? (Hone in on each)
    - In a world with no limitations, what would you change to CG?

5. Other general questions:
    - Age
    - Hobbies
    - Choice of pseudonym
